# Investigating Viral Involvement in Immunocompromised Patients Using Comprehensive Infectious Disease Testing Including FilmArray Respiratory Panel 2.1 on Bronchoscopy: A Retrospective Study

**DOI:** 10.7759/cureus.38820

**Published:** 2023-05-10

**Authors:** Tomoaki Nakamura, Ryosuke Imai, Atsushi Kitamura, Clara So, Shosei Ro, Kohei Okafuji, Yutaka Tomishima, Torahiko Jinta, Naoki Nishimura

**Affiliations:** 1 Department of Pulmonary Medicine, Thoracic Center, St. Luke's International Hospital, Tokyo, JPN

**Keywords:** infectious disease diagnosis, covid-19 pandemic, immunocompromised patients, bronchoalveolar lavage (bal), filmarray respiratory panel 2.1

## Abstract

Introduction

Reports are rare on the usefulness of the FilmArray Respiratory Panel 2.1 (FARP) using lower respiratory tract specimens. This retrospective study assessed its use, as part of a comprehensive infectious disease panel, to detect the viral causes of pneumonia using bronchoalveolar lavage samples from immunosuppressed patients.

Methods

This study included immunocompromised patients who underwent bronchoalveolar lavage or bronchial washing by bronchoscopy between April 1, 2021, and April 30, 2022. The collected samples were submitted for comprehensive testing, including FARP test; reverse transcription polymerase chain reaction (RT-PCR) for cytomegalovirus, varicella-zoster virus DNA, and herpes simplex virus; PCR for *Pneumocystis jirovecii* DNA; antigen testing for *Aspergillus* and* Cryptococcus neoformans*; and loop-mediated isothermal amplification method for* Legionella*.

Results

Out of 23 patients, 16 (70%) showed bilateral infiltrative shadows on computed tomography and three (13%) were intubated. The most common causes of immunosuppression were anticancer drug use (n=12, 52%) and hematologic tumors (n=11, 48%). Only two (9%) patients tested positive for severe acute respiratory syndrome coronavirus 2 and adenovirus by FARP. Four patients (17%) tested positive for cytomegalovirus by RT-PCR, but no inclusion bodies were identified cytologically. Nine (39%) patients tested positive for *Pneumocystis jirovecii* by PCR, but cytology confirmed the organism in only one case.

Conclusions

Comprehensive infectious disease testing, performed using bronchoalveolar lavage samples collected from lung lesions in immunosuppressed patients, showed low positive detection by FARP. The viruses currently detectable by FARP may be less involved in viral pneumonia diagnosed in immunocompromised patients.

## Introduction

The FilmArray respiratory panel was originally developed for pediatric patients and used nasopharyngeal swab specimens for the highly sensitive detection of viruses that cause upper respiratory tract infections and for bacteria that are difficult to culture [1-5]. It has been reported to be faster, simpler, and more sensitive than conventional methods, like individual PCR, direct fluorescent antibody, enzyme immunoassay, and virus culture methods [6]. The bioMérieux FilmArray respiratory panel 2.1 (FARP), an improved version, can also detect severe acute respiratory syndrome coronavirus 2 (SARS-CoV-2), responsible for the recent COVID-19 pandemic [7]. In total, FARP can detect 22 pathogens, including 18 viruses and four bacteria. For all microorganisms, positive percent agreement and negative percent agreement were 66.7-100% and 93.5-100%, respectively, compared to reference methods [8,9]. Since FARP was approved for manufacturing and marketing in Japan in June 2020, clinical studies on FARP using nasopharyngeal swab specimens have increased [10,11].

Immunosuppressed patients showed more FARP-positive results than non-immunosuppressed patients tested with earlier versions of the FARP, and bronchoalveolar lavage (BAL) specimens were more sensitive than nasopharyngeal swab specimens [12-14]. Therefore, more FARP-positive results were predicted in lower respiratory tract specimens from immunocompromised patients, which could be predicted to be clinically valid. However, these reports pre-dated the COVID-19 pandemic and did not use the newer version of FARP. Therefore, different results could have been obtained under the COVID-19 pandemic.

We performed BAL in almost all immunosuppressed patients with undiagnosed lung lesions. To aid in diagnosis, we routinely performed a fixed panel of tests, including FARP, contextually referred to as ‘comprehensive infectious disease testing’. In this study, a retrospective analysis of the results of comprehensive infectious disease testing was conducted to assess the usefulness of FARP in detecting the presence of pathogens in BAL specimens.

## Materials and methods

Study design

A retrospective observational study was conducted in our hospital between April 1, 2021, and April 30, 2022. The study included consecutive cases in which BAL or bronchial washing was performed to collect samples from pulmonary lesions in immunosuppressed patients. The collected samples were submitted for comprehensive testing, including FARP test; reverse transcription polymerase chain reaction (RT-PCR) for cytomegalovirus, varicella-zoster virus DNA, and herpes simplex virus; PCR for *Pneumocystis jirovecii* DNA; antigen testing for *Aspergillus* and *Cryptococcus neoformans*; and loop-mediated isothermal amplification method for *Legionella*. No specific exclusion criteria were applied.

In addition to patient characteristics, computed tomography (CT) findings, BAL results, and other bronchoscopy results were collected retrospectively. The selection of cases for comprehensive infectious disease testing, including FARP, and the content of bronchoscopy were determined in consensus with a senior fellow of the Japanese Respiratory Society and the Japan Society for Respiratory Endoscopy. The final diagnosis was determined based on the results of bronchoscopy with other clinical symptoms, blood test results, and CT imaging findings.

According to the Infectious Disease Society of America 2013 guidelines, immunocompromised patients are defined as those either having an ongoing hematologic malignancy, neutropenia, or steroid-sparing immunosuppressive therapy [6,15,16]. This retrospective study was approved by the Institutional Review Board of St. Luke’s International Hospital (no. 22-R047). The requirement for informed consent was waived by the Institutional Review Board of St. Luke’s International Hospital owing to the retrospective study design. This study was conducted in accordance with the principles of the Declaration of Helsinki.

Bronchoscopy

Infectious disease screening tests were performed using BAL samples from 22 (96%) patients and bronchial lavage samples from one (4%) patient. BAL was performed in the middle lobe or lingula division in 18 (82%) patients with a median collection volume of 93 ml. Before other procedure using bronchoscopies, 150 ml BAL was performed in three 50 ml aliquots. Only one case used 100 ml. BAL fluid (BALF) was sent for cytology and microbiological analysis. No complications were observed during bronchoscopy.

Statistical analysis

All statistical analyses were performed using EZR (Saitama Medical Center, Jichi Medical University, Saitama, Japan) [17], which is a graphical user interface for R Version 4.1.0 (The R Foundation for Statistical Computing, Vienna, Austria). Continuous variables are summarized using mean (range) and categorical variables using frequency (%). Positive results for comprehensive infectious disease tests were also summarized using 95% confidence intervals (CI).

## Results

In this study, 23 immunocompromised patients undergoing bronchoscopy were tested using comprehensive infectious disease screening, including FARP. Bilateral infiltrative shadows were found on CT of 16 (70%) patients and three (13%) were intubated. The most common causes of immunosuppression were anticancer drug use and hematologic tumors in 12 (52%) and 11 (48%) patients, respectively. Two (9%) patients underwent hematopoietic stem cell transplantation (HSCT) (Table 1). In most cases, a comprehensive infectious disease testing was submitted by using BALF, and the BALF recovery rate was good (Table 2).

**Table 1:** Patient and disease characteristics CT, computed tomography; HIV, human immunodeficiency virus; HSCT, hematopoietic stem cell transplantation; SOT, solid organ transplantation *Cumulative total

Patient and disease characteristics	Total (n=23)
Age, median (range), years	66 (35-85)
Males, n (%)	17 (74)
Oxygen therapy, n (%)	
Intubation	3 (13)
Nasal cannula	8 (35)
Room air	12 (52)
Antibiotic therapy during bronchoscopy, n (%)	
Antibacterial agent	13 (57)
Antifungal agent	5 (22)
Antiviral agent	4 (17)
CT findings of the lesion during bronchoscopy, n (%)	
Bilateral infiltration shadow	16 (70)
Patchy shadow	4 (17)
Nodules	3 (13)
Condition leading to immunosuppression*	
Chemotherapy	12 (52)
Hematologic disease	11 (48)
Immunosuppressant therapy	6 (26)
Steroid therapy	3 (13)
Neutropenia	3 (13)
HIV infection	1 (4)
Comorbidity, n (%)	
Hematologic disease	11 (48)
Lymphoma/leukemia	7 (31)/4 (17)
HSCT	2 (9)
Solid tumors	4 (17)
SOT	0 (0)
Collagen Disease	3 (13)

**Table 2:** Procedural details of bronchoscopy BAL, bronchoalveolar lavage; BALF, bronchoalveolar lavage fluid *23 patients undergoing bronchoscopy †22 patients undergoing BAL

Procedural details of bronchoscopy	Total
Diagnostic procedure, n (%)*	
BAL	22 (96)
Bronchial washing	1 (4)
Brush	6 (26)
Transbronchial biopsy	5 (22)
Transbronchial lung biopsy	3 (13)
Transbronchial lung cryobiopsy	1 (4)
Site of BAL performed, n (%)†	
Right upper lobe	2 (9)
Right middle lobe	16 (73)
Right lower lobe	1 (5)
Left upper lobe	1 (5)
Lingula division	2 (9)
The volume of recovered BALF, median (range), mL	93 (40-114)

FARP showed positive test results for SARS-CoV-2 and adenovirus in two cases (9%, 95% CI 1.1-28%), and the SARS-CoV-2-positive case was an overlap infection with Pneumocystis jirovecii pneumonia (PCP). Adenovirus-positive cases were diagnosed as organizing pneumonia based on CT shadows and a clinical course. Bacterial cultures were positive in six cases, and all acid-fast Bacillus cultures were negative. Cytomegalovirus (CMV) RT-PCR was positive in four cases (17%, 95% CI 5-38.8%), but no inclusion bodies were identified by cytology. *Pneumocystis jirovecii* was positive in nine cases (39%, 95% CI 19.7-61.5%), but cytology confirmed the organism in only one case. The *Aspergillus* antigen was positive in one case. All other tests were negative (Table 3).

**Table 3:** Pathogens identified by infectious disease screening tests FARP, FilmArray respiratory panel 2.1; PCR, polymerase chain reaction; RT-PCR, reverse transcription polymerase chain reaction; CMV, cytomegalovirus; VZV, varicella zoster virus; HSV, herpes simplex virus; LAMP, loop-mediated isothermal amplification; CI, confidence interval * Only Stenotrophomonas maltophilia was identified as the causative organism. Coagulase-negative Staphylococcus, β-Streptococcus sp., yeast sp., Candida albicans, Klebsiella pneumoniae, and Stenotrophomonas maltophilia were detected. †One patient was determined to be truly infected. ‡Seven patients were determined to be truly infected.

Pathogens identified by infectious disease screening tests	Total (n=23)	95% CI (%)
Bacterial culture*, n (%)	6 (26)	10.2–48.4
Acid-fast bacilli culture, n (%)	0 (0)	0.0–14.8
Virus	6 (26)	10.2–48.4
FARP, n (%)	2 (9)	1.1–28
SARS-CoV-2, n (%)	1 (4)	0.1–21.9
Adenovirus, n (%)	1 (4)	0.1–21.9
CMV RT-PCR^†^, n (%)	4 (17)	5.0–38.8
VZV PCR, n (%)	0 (0)	0.0–14.8
HSV RT-PCR, n (%)	0 (0)	0.0–14.8
Pneumocystis jirovecii PCR^‡^, n (%)	9 (39)	19.7–61.5
Aspergillus antigen, n (%)	1 (4)	0.1–21.9
Legionella LAMP method, n (%)	0 (0)	0.0–14.8
Cryptococcus neoformans antigen, n (%)	0 (0)	0.0–14.8

The most common final diagnoses of pulmonary lesions were drug-induced lung disease and PCP in seven cases each. Organizing pneumonia was diagnosed in three cases (13%), and CMV pneumonia was diagnosed in one case (4%) (Table 4).

**Table 4:** Final diagnosis of lung lesions

Final diagnosis of lung lesions	Total (n=23)
Drug-induced pneumonitis, n (%)	7 (30)
Pneumocystis carinii pneumonia, n (%)	7 (30)
Co-infection of COVID-19	1 (4)
Organizing pneumonia, n (%)	3 (13)
Diffuse alveolar hemorrhage, n (%)	2 (9)
Cytomegalovirus pneumonia, n (%)	1 (4)
Hypersensitivity pneumonitis, n (%)	1 (4)
Relapse of angioimmunoblastic T-cell lymphoma, n (%)	1 (4)
*Aspergillosis* and *Stenotrophomonas maltophilia* pneumonia n (%)	1 (4)

## Discussion

This retrospective study reviewed the results of comprehensive infectious disease testing, including FARP, using BAL specimens from undiagnosed lung lesions in 23 immunosuppressed patients. FARP results were positive for SARS-CoV-2 and adenovirus in only two cases.

The positive results of the earlier version of the FilmArray respiratory panel, performed relatively recently with nasopharyngeal swabs from pediatric patients, were as high as 64-80% [18,19]. Meanwhile, in adult patients, the positive results of the FilmArray respiratory panel have been reported to decrease with increasing age [20]. Positive results have been reported in immunocompromised more frequently than in immunocompetent patients [14]. Attempts are also being made to submit FARP using BAL samples rather than traditional nasopharyngeal swabs. In studies wherein BAL was performed in immunocompromised adults using earlier versions of the FARP, the positive results ranged from 24-31% [12,13]. These studies also suggested a benefit to submitting BAL specimens, as some cases were negative when testing nasopharyngeal swabs, but positive when testing BAL samples. All studies presented so far had been conducted prior to the COVID-19 pandemic. During the COVID-19 pandemic, there was a significant decrease in the number of influenza infections and other respiratory infections compared with that in previous years [21-24]. The FARP has been updated, and the latest version of the FARP is now capable of detecting SARS-CoV-2 [7]. Consequently, pre-pandemic study results may not apply to current conditions. Since no studies have submitted and reviewed FARP in BAL specimens, it was significant to provide additional study results.

The FARP-positive results in this study are lower than the results in previous studies, which ranged from 24% to 31%. There are several possible reasons for this finding. First, these previous studies included HSCT and solid organ transplantation as causes of immunodeficiency in 50-70% of the cases. The predominant causes of immunodeficiency in this study were the use of anticancer drugs and hematologic tumors, with HSCT being the cause in only 9% of the cases. Given prior studies showing a trend toward fewer positive results using FARP in immunocompetent patients, it is possible that the low incidence of positive results in this study [14] could have resulted from our patients being in a less severe immunosuppressed state.

A second reason for the low incidence of positive results could be the infection prevention behaviors adopted during the COVID-19 pandemic. Since the pandemic, a decline in influenza and other respiratory infections has been reported in many parts of the world [21-24]. Possible reasons for this include non-pharmaceutical interventions, such as physical distancing, isolation of symptomatic individuals, school closures, and the use of face masks [24,25]. Particularly in Japan, public precautions are still being taken, including the use of masks during short-distance conversations. Consequently, it is possible that the types of respiratory infections have changed, leading to the low incidence of positive results in our study.

There was some viral involvement in the pneumonia cases in our study group, including one patient diagnosed with CMV pneumonia. Furthermore, our study included many patients who were positive for *Pneumocystis jirovecii* PCR and CMV RT-PCR, as reported previously [26,27]. FARP does not include these pathogens, making it unsuitable as a panel test for pneumonia in immunocompromised patients. Therefore, a new test panel must be developed for immunocompromised patients.

This study has some limitations. Because the patient population was selected based on actual clinical practice, other studies may obtain different results if their patient population is different or controlled. In addition, false-negative test results, especially for bacterial cultures, may be due to prior antimicrobial therapy in more than half of the patients.

## Conclusions

When comprehensive infectious disease testing was performed on BAL specimens from lung lesions in immunosuppressed patients, the incidence of positive results of FARP was low. Viruses detectable using FARP do not appear to be the primary causes of pneumonia in immunocompromised adult patients.
